# Detecting Changes in Comfort, Pain, and Mobility Over Clinical Milestones for Individuals With Lower Limb Loss

**DOI:** 10.33137/cpoj.v7i1.43890

**Published:** 2024-10-25

**Authors:** B.M Pousett, C.C Harasym, M.S Rapaport, T Richardson, J Spellen, D.W Moe, W.C Miller

**Affiliations:** 1 Barber Prosthetics Clinic, Vancouver, Canada.; 2 Rehabilitation Sciences, Faculty of Medicine, University of British Columbia, Vancouver, Canada.; 3 Biomechanical Engineering, University of British Columbia, Vancouver, Canada.; 4 G.F. Strong Rehabilitation Research Program & Department of Occupational Science and Therapy, Faculty of Medicine, University of British Columbia, Vancouver, Canada.

**Keywords:** Prosthesis, Amputation, Lower Limb Prosthesis, Outcome Measure, Socket Comfort, Mobility, Pain, Practice-Based Evidence, 2MWT, Functional Mobility, Rehabilitation, PLUS-M

## Abstract

**BACKGROUND:**

Functional mobility, comfort and the absence of pain are key goals of prosthetic treatment. Outcome measures (OMs) evaluate the impact of treatment and normative and minimal detectable change (MDC) values are key to interpreting these scores and measuring treatment outcomes.

**OBJECTIVES:**

This study seeks to 1) present practice-based normative values of four commonly used OMs at four prosthetic milestones and 2) explore the MDC of the measures over the treatment period.

**METHODOLOGY:**

A chart review was conducted of OMs collected with individuals with lower limb loss between January 1, 2015, and December 31, 2023. This included data for individuals with unilateral transtibial (TT), transfemoral (TF) and rotationplasty (RP) amputations and bilateral transtibial amputation (BTT). OMs included the Socket Comfort Score (SCS), Pain Scale (PS), 2 Minute Walk Test (2MWT), and Prosthetic Limb Users Survey of Mobility (PLUS-M). Data were collected at four milestone time points: (1) Baseline and (2) Discharge from Rehabilitation for those in initial prosthetic rehabilitation, and (3) Initial Evaluation and (4) Definitive Delivery for those receiving a replacement socket. Normative values and MDC values were calculated.

**FINDINGS:**

Data from 30 individuals undergoing in-patient rehabilitation and 74 individuals receiving a replacement socket were included. Practice-based normative data were different for each level of amputation and milestone and had the following ranges: SCS: 5.7 – 9.1, PS: 0.8 – 3.7, 2MWT: 68.4 – 146.3 m and PLUS-M: 38.9 – 57.3. MDC values also varied based on time in treatment (Rehabilitation: SCS = 2.5, PS = 1.6, 2MWT = 32.6, PLUS-M = 8.8; Replacement Socket: SCS = 3.1, PS = 2.6, 2MWT = 38.9, PLUS-M = 4.0). All measures had a statistically significant change over the intervention, however, no average scores changed by greater than the MDC.

**CONCLUSIONS:**

The normative data and MDC scores demonstrate the PS & PLUS-M are useful measures of pain and mobility at all points within treatment. The 2MWT is indicated for individuals in rehabilitation, while the SCS is indicated for those receiving a replacement socket, as both effectively measure treatment goals that are particularly important for each phase of rehabilitation. This provides clinicians with practice-based evidence that enables them to interpret OM scores, a critical part of the decision-making process along the treatment journey.

## INTRODUCTION

Prosthetic treatment seeks to achieve a shared goal and influence a change in function. Mobility is a primary goal of treatment and is supported by a comfortable prosthesis that is free of pain.^1^ The provision of a prosthesis occurs at two phases: initial rehabilitation and subsequent replacement socket.

The baseline and end point of each phase are important milestones, and progress should be measured at these points to determine if a change has occurred and a goal has been achieved. Socket comfort, pain and mobility are critical indicators of successful prosthetic treatment. However, there is little data regarding how these values change at different clinical milestones.

Outcome measures (OMs) are a way to provide evidence of change toward a goal.^2–4^ The 2 Minute Walk Test (2MWT),^5^ Prosthetic Limb Users Survey of Mobility (PLUS-M),^6^ Socket Comfort Score (SCS),^7^ and Pain Scale (PS)^8^ are four OMs that measure critical prosthetic treatment indicators. The Canadian Amputations Rehabilitation Evidenced-Based Review Group^9,10^ and the American Academy of Orthotists and Prosthetists^11^ recommend these measures and their constructs as they capture important and relevant information and are easy to integrate into the clinic environment. However, to be useful, OM scores need to have meaning.

One form of meaning is normative values. Normative values, the average values for a population, are dependent on population demographics (e.g., age, sex, etc.) and pathologies (e.g., cause of amputation) and allow the clinician to compare the patient’s outcome with others in the population.^12^ Normative values are often collected in research settings, where population characteristics and administration methods may differ from clinical environments.^13^ Practice-based normative values provide evidence based in clinical practice. Practice-based normative values are similar to the clinical population and are also recorded in the clinical setting. The Minimal Detectable Change (MDC) of a measure is the smallest change that falls outside of measurement error.^12^ This is crucial for measuring treatment outcomes as if a patient’s OM score has changed by greater than the MDC, it indicates that they have experienced a true change.^14^

While some OMs have interpretability values from research and clinical settings (e.g., the 2MWT),^15–17^ many studies do not specify the timepoint within the treatment pathway and cannot show how an OM changes as the individual progresses. The relationship between OM scores differs at different milestones,^18^ and we expect that the practice-based normative values and changes in measures will be different at different milestones as well. For example, decreased functional mobility is a common consequence of lower limb loss^19^ that rehab programs seek to address. We hypothesized those going through initial prosthetic rehabilitation may have a larger change in the OMs measuring functional mobility, as demonstrated by increased distance walked in the 2MWT. Without clinical values to give meaning to OM scores and their changes, the intended purposes for using OMs cannot be realized.^4,13^

Our research objectives were to: **A.** Present practice-based evidence of the normative values of four commonly used OMs at four milestones: (1) Baseline and (2) Discharge from Rehabilitation for individuals in initial prosthetic rehabilitation, and (3) Initial Evaluation and (4) Definitive Delivery for those receiving a replacement socket; **B.** Explore which OMs scores changed by more than the MDC over the treatment period and may be useful for measuring the effectiveness of prosthetic treatment interventions.

## METHODOLOGY

### Study Design & Sample

A chart review of patients who had OMs data recorded at Barber Prosthetics Clinic between January 1, 2015, and December 31, 2023, was conducted. The study was approved by the Clinical Research Ethics Board at the University of British Columbia (H21-02131; H24-00501). To be included, patients had to be over the age of 18, have unilateral or bilateral amputations above the ankle and below the hip, and have OMs data recorded in their chart. Patients were excluded if they did not have OMs data for at least one OM at Baseline and Discharge from Rehabilitation, or Initial Evaluation and Definitive Fitting.

**Figure 1:**
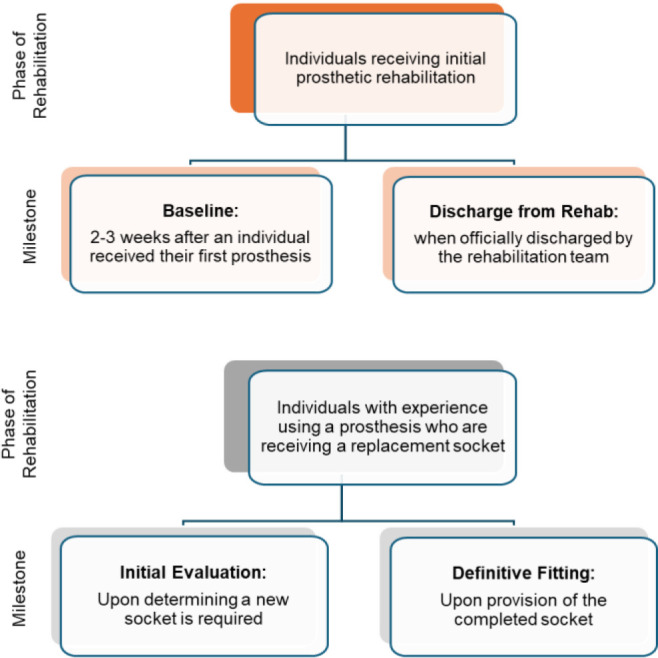
Definitions of the prosthetic milestones included in the two distinct phases of the treatment journey.

Data were captured at two clinical milestones for each of the two distinct phases of the treatment journey(**Figure 1**). Initial prosthetic rehabilitation is a distinct period from the rest of the prosthetic journey due to its large learning component and rapid changes in socket fit and mobility.

### Outcome Measures:

The OMs were administered by Certified Prosthetists who had completed computer-based training to use these four specific OMs^20^ as well as in-person feedback to ensure consistency in administration. The SCS, PS, PLUS-M and 2MWT were administered at clinical milestones, regardless of treatment goals.

The 2MWT is a performance-based measure of aerobic capacity and functional mobility. It records how many meters an individual can walk in 2 minutes. A 10 m hallway with in-floor markings was used in most instances but walkways of different lengths were used when 10 m hallways were unavailable. The variability in hallways length is a known issue in the clinic use of the 2MWT.^13^ Assistive devices were used if needed. The 2MWT has published normative values based on age, sex, cause of amputation, and level of amputation which makes it easy to interpret to the context of the patient.^5,15,21-24^ It is the only measure that has values specified at clinical milestones, as one study presented scores at Discharge from Rehabilitation and a later follow-up.^16^ The measure has test-retest reliability for individuals with limb loss, ICC = 0.83, and an MDC of 34.4 m.^24^

The PLUS-M is a 12 item self-report mobility survey that asks individuals with limb loss to rate their ability to complete tasks on a 5-point scale ranging from without any difficulty [5] to unable to do [1].^25^ Clinicians read aloud the survey for patient who struggled to complete the test independently. The PLUS-M is scored by converting the raw scores to a T-score, where a T-score of 50 relates to the average score of the original development sample.^25^ Test-retest reliability has ICC=0.96, and the MDC-90 of the t-score of 4.50.^26^

The SCS and PS are self-report measures of socket comfort and pain intensity, respectively. They are both rated verbally on a numerical 11-point scale from 0 (SCS: least comfortable socket they can imagine, or PS: no pain) to 10 (SCS: most comfortable socket they can imagine, and PS: worst pain imaginable). The test-retest reliability of the SCS is ICC=0.77 and the MDC is 2.82.^26^

The PS, while previously used with people with amputations^27^ has little specific interpretability data published to date. A study with a combined sample of individuals with amputations and spinal cord injuries found a change of 1.8 corresponded to a meaningful change in pain.^27^ This measure is similar to the Patient-Reported Outcomes Measurement Information System 29-item profile (PROMIS-29) Pain Intensity, which asks the identical question but has instructions that suggest a different time period (7 days vs 24 hours).^8,28^ The PROMIS-29 Pain Intensity has test-retest reliability of ICC = 0.87 and an MDC of 1.97. We used the PS due to its clinical prominence, and as the PROMIS-29 represents the best available evidence, we relied on it to inform our interpretation of the PS.^26^

### Data Collection:

OMs data, treatment milestones, and demographic data including level of amputation, cause of amputation, time since amputation, sex, and date of birth were extracted from medical records. If a patient reached a milestone multiple times within the included dates (i.e., they had more than one replacement socket), the most recent milestone was used. Patient charts were included if they had data for at least one of the OMs at either Baseline and Discharge from Rehabilitation, or Initial Evaluation and Definitive Fitting, so that change scores could be calculated.

### Analysis:

Analysis was done using R Studio (Posit Software, PBC). Demographic data were analyzed using descriptive statistics and partitioned by level of amputation and phase of the treatment journey. Practice-based normative values for each measure were partitioned by treatment milestone. Mean changes in the OM scores over the paired milestones were presented. The mean change values were compared to calculated MDC values which were calculated using the following formula, using previously established ICC values.


$$MDC=1.645^{*}{\sqrt{2}}^{*}\;SEM$$

$$SEM={\text{standardDeviation}}_{BaselineScores}\sqrt{\left(1-ICC_{test-retest}\right)}$$

All MDC values use a 90% confidence interval to be consist with what is reported in the literature.^24^

The significance of the mean change over treatment was calculated using a 95% confidence interval (a = 0.05). A beneficial change for the patient would include a positive increase in SCS, PLUS-M and 2MWT by greater than the MDC, along with a negative change in the PS by more than the MDC.

## RESULTS

Data were collected from 30 individuals going through initial prosthetic rehabilitation and 74 individuals getting a replacement socket (**Table 1**). Most individuals in rehabilitation were males with transtibial amputations, with a mean age of 65.7 years (SD = 12.9). Among those receiving a replacement socket, the majority were also male, with a mean age of 48.5 years (SD = 16.8), and had amputations due to various causes. The subgroup with rotationplasty amputations was mostly female, with a mean age of 29.3 years (SD = 5.4).

**Table 1:** Demographic information of individuals included.

Individuals with Transtibial Amputations		
	Receiving Initial Prostdetic Rehabilitation (N = 27)	Receiving a Replacement Socket (N = 53)
Age (years) – Mean (SD)	65.8 (13.6)	49.9 (16.0)
Male – Number (%)	18 (66.7%)	43 (81.1%)
Etiology – Number (%)		
Cancer/Tumor	1 (3.7%)	5 (9.4%)
Congenital	1 (3.7%)	2 (3.8%)
Injury/Trauma	2 (7.4%)	23 (43.4%)
Vascular/Diabetes	22 (81.5%)	21 (39.6%)
Unknown/Other	1 (3.7%)	2 (3.8%)
Time Since Amputation (years) – Mean (SD)	0.5 (1.0)	9.1 (12.0)
Time in Rehab (weeks) – Mean (SD)	6.4 (4.5)	N/A
Individuals with Transfemoral Amputations		
	Receiving Initial Prosthetic Rehabilitation (N = 3)	Receiving a Replacement Socket (N = 10)
Age (years) – Mean (SD)	65.0 (3.6)	51.0 (22.3)
Male – Number (%)	2 (66.7%)	8 (80.0%)
Etiology – Number (%)		
Cancer/Tumor	–	3 (30.0%)
Congenital	–	1 (10.0%)
Injury/Trauma	–	4 (40.0%)
Vascular/Diabetes	3 (100.0%)	2 (20.0%)
Time Since Amputation (years) – Mean (SD)	0.4 (1.0)	22.2 (14.2)
Time in Rehab (weeks) – Mean (SD)	7.4 (4.2)	N/A
Individuals with Rotationplasty Amputations		
	Receiving Initial Prosthetic Rehabilitation (N = 0)	Receiving a Replacement Socket (N = 4)
Age (years) – Mean (SD)	–	29.2 (5.3)
Male – Number (%)	–	1 (25.0%)
Etiology – Number (%)		
Cancer/Tumor	–	1 (25.0%)
Congenital	–	2 (50.0%)
Unknown	–	1 (25.0%)
Time Since Amputation (years) – Mean (SD)	–	20.0 (10.9)
Individuals with Bilateral Transtibial Amputations		
	Receiving Initial Prosthetic Rehabilitation (N = 0)	Receiving a Replacement Socket (N = 7)
Age (years) – Mean (SD)	–	45.6 (12.8)
Male – Number (%)	–	5 (71.4%)
Etiology – Number (%)		
Injury/Trauma	–	2 (28.6%)
Vascular/Diabetes	–	2 (28.6%)
Unknown	–	3 (42.9%)
Time Since Amputation (years) – Mean (SD)	–	4.0 (3.3)

Note: Time since amputation for those receiving initial prosthetic rehabilitation was the time between the discharge from rehab appointment and their amputation date. time since amputation for those receiving a replacement socket was the time between the definitive delivery appointment and their amputation date.

### Practice-Based Normative Values

Practice-based normative values are presented in **Table 2**. The numbers of individuals included for each measure range and some measures are marked as N/A because it is not always clinically appropriate to administer every measure. For individuals in rehabilitation, males were found to have higher SCS and lower PS at Baseline and higher PLUS-Ms scores at both Baseline and Discharge from Rehabilitation. However, for individuals receiving a replacement socket, there is no difference in scores across sexes at either milestone.

**Table 2:** Clinical normative data for outcome measures used in prosthetic treatment.

	SCS	2MWT (m)	PS	PLUS-M
**Individuals Receiving Initial Prosthetic Rehabilitation**				
**Baseline**				
Transtibial (N = 24, 20, 17, 26) – Mean (SD)	7.2 (2.3)	68.4 (32.4)	2.7 (1.9)	38.9 (19.4)
Transfemoral (N = 2, 0, 0, 2) – Mean (SD)	7.5 (0.7)	N/A	N/A	45.6 (4.0)
**Discharge from Rehabilitation**				
Transtibial (N = 24, 20, 17, 26) – Mean (SD)	8.5 (1.0)	107.4 (32.3)	1.0 (1.0)	53.4 (8.4)
Transfemoral (N = 2, 0, 0, 2) – Mean (SD)	8.5 (0.7)	N/A	N/A	43.7 (4.8)
**Individuals Receiving a Replacement Socket**				
**Initial Evaluation**				
Transtibial (N = 56, 50, 48, 56) – Mean (SD)	5.7 (2.9)	126.5 (36.4)	3.7 (3.2)	51.6 (9.1)
Transfemoral (N = 10, 12, 10, 12) – Mean (SD)	5.8 (2.8)	104.7 (53.8)	3.6 (3.1)	48.2 (6.4)
Transtibial, Bilateral (N = 8, 6, 5, 8) – Mean (SD)	7.3 (1.6)	132.3 (48.2)	2.5 (1.3)	53.0 (6.6)
Rotationplasty (N = 3, 3, 2, 4) – Mean (SD)	8.0 (0.0)	100.7 (38.2)	1.5 (0.7)	57.2 (11.0)
**Definitive Fitting**				
Transtibial, (N = 56, 50, 48, 56) – Mean (SD)	8.9 (1.2)	132.6 (41.4)	1.4 (1.7)	53.2 (11.4)
Transfemoral (N = 10, 12, 10, 12) – Mean (SD)	8.0 (2.9)	107.6 (42.2)	3.1 (3.5)	51.2 (9.3)
Transtibial, Bilateral (N = 8, 6, 5, 8) – Mean (SD)	9.1 (0.8)	146.3 (56.4)	0.8 (1.0)	52.9 (8.0)
Rotationplasty (N = 3, 3, 2, 4) – Mean (SD)	9.0 (1.0)	112.7 (46.5)	1.5 (0.7)	57.3 (9.7)

Note: The N values are listed as (NSCS, N2MWT, NPS, NPLUS-M) and provide the number of participants included in the SCS, 2MWT, PS and PLUS-M calculations respectively.

### Changes in Scores over Treatment Interventions

Average changes in scores and the percentage of scores that changed by more than the MDC are presented in **Table 3**. For individuals in rehabilitation, the PS & 2MWT measured change most often, and the SCS measuring change the least often. For individuals receiving a replacement socket, the PLUS-M measured changes most often while the 2MWT rarely did.

**Table 3:** Average changes in scores for individuals with lower limb loss over prosthetic treatment.

N	Change in Score – Mean (SD) (m)	Significance – p-value (0.05)	MDC - 90 Calculated	% Changed by MDC Calculated
**Individuals Receiving Initial Prosthetic**				
SCS (N = 26)	1.3 (2.4)	0.013^*^	2.5	19%
2MWT (N = 21)	37.9 (30.0)	<0.000^*^	32.6	48%
PS (N = 18)	−1.2 (2.4)	0.053	1.6	50%
PLUS-M (N = 28)	13.3 (18.3)	<0.000^*^	8.8	43%
**Individuals Receiving a Replacement Socket**				
SCS (N = 68)	2.9 (2.9)	<0.000^*^	3.1	39%
2MWT (N = 62)	6.3 (18.6)	0.009^*^	38.9	6%
PS (N = 59)	−1.9 (2.9)	<0.000^*^	2.6	31%
PLUS-M (N = 70)	2.3 (5.6)	<0.00^*^	4.0	47%

* Indicates change in score is significant from before to after treatment using a = 0.05.

## DISCUSSION

Through analyzing data from individuals in prosthetic rehabilitation or receiving a replacement socket, we were able to provide practice-based normative values for four commonly used OMs (SCS, PS, 2MWT & PLUS-M) based on level of amputation at four distinct clinical milestones. We were also able to provide practice-based MDC scores as well as demonstrate which measures recorded a change at which treatment milestones. This information provides clinicians valuable information about interpreting OM scores at these clinical milestones. As each outcome measure had a different purpose, it is often advantageous to use a variety of OMs to measure the changes in distinct aspects of the individuals experience (e.g. comfort and functional mobility). The practice-based normative values, mean changes in scores, and calculated MDC values are different for each measure at the different milestones, highlighting the importance of interpreting the data within these strata and the novelty of this approach. When comparing calculated MDC values to those published, the calculated values are often smaller for those in rehabilitation and greater for those receiving a replacement socket. This value is related to the homogeneity of the scores for our specific populations, with our population having less variability in OM scores at Baseline for those in rehabilitation and more variability in OM scores at Initial Evaluation for those receiving a replacement socket.

The demographics were different for the two segments of the treatment journey, further highlighting the difference in new patients seen in rehabilitation hospitals and experienced patients seen in clinics. Those in rehabilitation are older and have amputations largely due to vascular reasons while those receiving a replacement socket are younger and have a wider variety of etiologies of amputation.

### SCS

For individuals in rehabilitation, SCS were high at Baseline and remained high at Discharge from Rehabilitation. Because the scores remained constant, the average change in SCS did not change by the MDC and individuals experienced change greater than MDC only 19% of the time. This is likely because in this specific clinical environment, patients typically see their prosthetist at least once a week for any adjustments to ensure the socket is comfortable.

Between Initial Evaluation and Definitive Fitting, average change in SCS scores were just below the MDC and a change greater than the MDC was experienced by 40% of individuals. Limb volume changes are a common clinical reason for replacement sockets, leading to uncomfortable limb-socket interfaces and lower SCS at Initial Evaluation. SCS were high when individuals received their definitive prostheses, suggesting the goal of providing a comfortable interface had been achieved.

While transtibial and transfemoral prosthesis users reported similar levels of comfort at Initial Evaluation, transfemoral prosthesis users reported lower levels of comfort at the Definitive Fitting. This supports previous research which found that individuals with transfemoral amputations experience diminished comfort and functional outcomes than those with other levels of amputations.^29^ The nature of the intimacy of the socket fit could be a reason transfemoral users have less comfortable fitting sockets. In addition, individuals using rotationplasty prostheses had high levels of comfort at both time points. This is hypothesized to be because the deterioration of external prosthetic joints and the need for a new socket with new joints is a common clinical reason for receiving a replacement socket and does not reflect in comfort scores. Previous studies found average SCS to be 4.8 before receiving a new socket, 6.8 twenty minutes after receiving a new diagnostic socket, and 8.4 for individuals who had been using a well-fitting socket for 6 months to 5 years, based on a sample of various lower extremity amputation.^7^ The current sample had higher socket comfort scores at all time points, which may be due to having fewer transfemoral users, a younger average age, and a wider variety of amputation etiologies.

The SCS is suited for use with patients receiving a replacement socket, as it is easy to use, aligns with a common treatment goal, and frequently captures the impact the intervention has at this milestone. It also can be useful for guiding treatment decisions and adding clarity to the communication between patients and their prosthetists.^18^ For those in rehabilitation, while using the SCS in this setting can facilitate clinical conversation, it may not be as appropriate for measuring the impact of this intervention.

### PS

For individuals in rehabilitation, PS scores were low at Baseline and remained low at Discharge from Rehabilitation, indicating an absence of pain. PS values negatively correlate with SCS values as the presence of comfort often correlates to the absence of pain.^18^ While the PS does not have previous normative values published, the values at Definitive Fitting were lower than the Pain Intensity scale from the PROMIS-29 which reported an average score of 3.3 for a population that was older, with higher levels of amputations and more vascular etiologies^30^ than the current sample.

For individuals receiving a replacement socket, pain levels decreased from Initial Evaluation to Definitive Fitting, inversely to SCS, again demonstrating the clinical goal has been met in these instances.

The PS is suitable for use with patients in rehabilitation and with patients receiving a replacement socket as it captured change over 50% and 31% of the time, respectively. The PS is easy to use, aligns with a common treatment goal and provides clinicians with helpful information.

### 2MWT

2MWT scores changed notably for the transtibial population as individuals progressed through their rehab and were able to walk further. 2MWT scores (68.3 m at Baseline in Rehabilitation and 107.4 m at Discharge from Rehabilitation) were notably higher than previously reported patients in rehabilitation (20.4 m for males and 22.5 m for females at baseline and 46.0 m for males and 29.1 m for females at discharge).^16^ The current sample had similar mean ages and male/female composition, with no difference in mean scores based on sex. However, Brooks et al. measured the baseline scores earlier in the fitting process and had a shorter average time from fitting to discharge which may have contributed to the lower scores.

In addition, individuals with bilateral transtibial limb loss had the highest 2MWT scores at Initial Evaluation and Definitive Delivery. Previous research has found that individuals with unilateral transtibial amputations tend to have higher 2MWT scores than those with bilateral transtibial amputations during rehabilitation^16^ and it was hypothesized this relationship would exist for replacement sockets as well. Perhaps the younger mean age of the individuals with bilateral amputations in our study led to their higher functional mobility.

For individuals in rehabilitation, the average change in score was larger than the MDC and the 2MWT measured change greater than MDC 48% of the time. This demonstrated that while this measure is an effective way to document changes in functional mobility during initial rehabilitation, preamputation activity and comorbidities, and not just the disease or rehabilitation intervention, impact aerobic capacity and functional mobility.^31^

For individuals receiving a replacement socket, our sample also had slightly higher 2MWT scores than most previous studies (mean scores 98.8 – 113.2),^15,21-23^ but less than the most recent study (mean score 154.3 m).^32^ Scores tend to be higher in samples with more traumatic and fewer vascular amputations, more distal amputation levels, and younger populations. Although it was hypothesized that a new, well-fitting socket would improve walking capacity, this was not the case as it only measured a change greater than MDC 6% of the time. This demonstrates that straight line walking capacity is not affected by this intervention. Perhaps a measure including functional tasks could be more suited to capture a change in this population. There are also personal and environmental considerations such as co-morbidities, cardiac function, and confidence that determine walking capacity.

The 2MWT is most useful for individuals in rehabilitation as it is easy to administer and measures the impact of prosthetic rehabilitation on a patient’s functional mobility, which is a primary goal of rehabilitation.^1^ For individuals receiving a replacement socket, the 2MWT is not recommended as it does not capture the change that this treatment has.

### PLUSM

For most individuals in rehabilitation, PLUS-M scores were below those of the original PLUS-M development sample, which is intuitive as they were early in their rehabilitation.^25^ PLUS-M Scores at Definitive Fitting were close to the PLUS-M development sample for individuals with transtibial and transfemoral amputations due to trauma, which was the most common cause of amputation reported in this subgroup of our sample.^25^ For individuals in rehabilitation, the average change in score was larger than the MDC and measured a change greater than MDC 43% of the time. For individuals receiving a replacement socket, the average change in PLUS-M score was below the MDC, but it did capture a change greater than MDC 47% of the time, demonstrating it is more effective at capturing changes in mobility for this population than the 2MWT was.

The PLUS-M is useful for providing a snapshot of an individual’s perceived mobility at a point in time and successfully captures the change that prosthetic treatment has on an individual at both parts of the treatment journey. Perhaps for those whose mobility is the primary goal or concern, such as during the aging process, the usefulness of this measure would be even greater.

### Limitations

The data were collected in a single clinical practice. For the individuals in the rehabilitation sample, the age, sex, and etiology of amputation reflect the incidence of amputation in Canada. However, less individuals with transfemoral amputations were represented in the current study than the incidence rates suggests.^33^ For those receiving a replacement socket, the current sample was younger, had less individuals with transfemoral amputations, and had more individuals represented with traumatic, cancer or congenital amputations.^33^ But, this helps to ensure that demographics reflect other groups at similar milestones.

It should be noted that individuals in rehabilitation with transfemoral limb loss and individuals with rotationplasty amputations receiving a new socket had small sample sizes and therefore are not representative of that population and should be interpreted as such.

However, it is the first study to our knowledge that provides evidence for using OMs with this rotationplasty population and has been kept in to provide initial evidence for this group. In addition, this is one of the first studies presenting information on the Pain Scale for use with people with amputations. The practice-based normative values can begin to form the evidence on this measure. However, the MDC values were calculated using information from the PROMIS-29 and should be cautiously interpreted.

Future work can address the psychometric properties of the PS. Finally, since OMs were not selected based on treatment goals, they may be measuring constructs that were not addressed in treatment. Future work should integrate both treatment milestones and treatment goals to provide the most accurate assessment of the ability of OMs to capture changes due to treatment.

## CONCLUSION

This study provides clinicians with detailed information on how to interpret scores in clinical environments. The impact of the treatment was measured before and after each intervention using different OMs, thus indicating the need to consider treatment milestones when choosing OMs for clinical use. Placing more responsibility on the clinician to select an OM based on the patient’s goal and milestones could provide more meaningful results The development and use of outcome measures that capture changes at specific treatment milestones and are easy for both patients and prosthetists to use are critical for the successful implementation of these measures.

## DECLARATION OF CONFLICTING INTERESTS

The authors have no conflicts of interest to disclose.

## AUTHORS CONTRIBUTION

- **Brittany Mae Pousett:** conceptualization, study design, data curation, formal analysis, project administration, & writing – original draft, review & editing.
- **Charlene C Harasym:** study design, data curation, formal analysis, & writing – review & editing.
- **Malena Sofia Rapaport:** conceptualization, study design, investigation & writing – review & editing.
- **Tessa Richardson:** investigation & writing – review & editing.
- **Jesse Spellen:** investigation & writing – review & editing.
- **David W Moe:** conceptualization, investigation & writing – review & editing.
- **William C Miller:** conceptualization, study design, investigation, supervision & writing – review & editing.

## SOURCES OF SUPPORT

Research reported in this article was not supported by any funding agency in the public, commercial, or not-for-profit sectors.
